# Supplementary material for: Learning a pairwise epigenomic and transcription factor binding association score across the human genome
Source: Bioinformatics. 2026 Jan 20;42(2):btag024. doi: 10.1093/bioinformatics/btag024 (PMC12910503; doi:10.1093/bioinformatics/btag024)
Supplement: btag024_Supplementary_Data [file btag024_supplementary_data.zip › supplementary_figures_and_method.pdf]

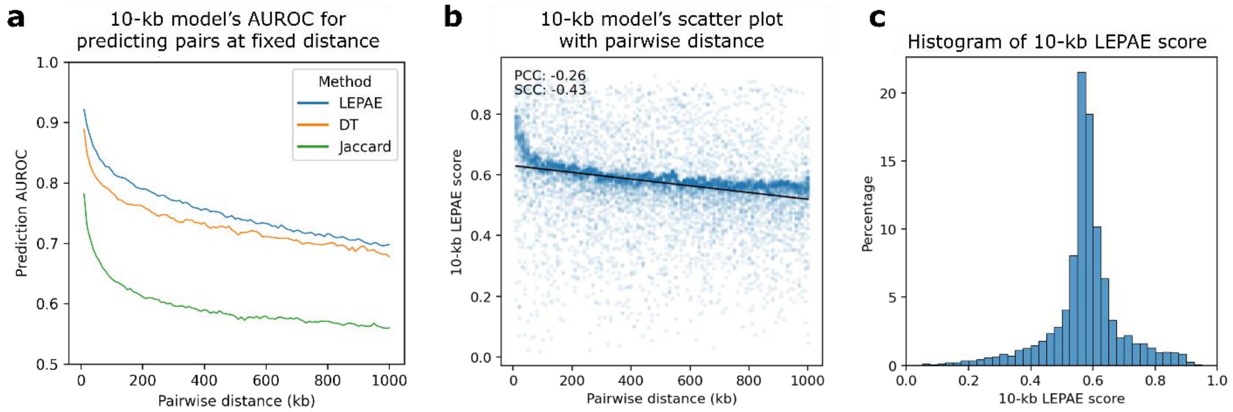

Supplementary Figure 1. Characteristics of the 10-kb LEPAE score.

a. Relationship between pairwise distance and prediction AUROC for 10-kb model. For each pairwise distance (x-axis), mean prediction AUROC of the 10-kb LEPAE score for distinguishing pairs of windows at that distance from randomly mismatched pairs of the same windows is shown in blue. The mean is computed from two sets of classifiers trained on non-overlapping training sets (**Methods**). Mean AUROC values when a decision tree (DT) instead of a neural network was used as the supervised classifier are shown in orange (**Supplementary Methods**). Mean AUROC values computed using Jaccard index instead of the LEPAE score to perform the same classification task are shown in green. Values belonging to the same method are connected by piecewise linear interpolation.

b. Scatter plot showing with a blue dot for each pair of windows the pairwise distance (x-axis) and the 10-kb LEPAE score (y-axis). Ten thousand random pairs are shown. A linear regression line fitted to the ten thousand random pairs is shown in black. Pearson correlation coefficient and Spearman correlation coefficient (PCC and SCC), computed from 1 million randomly selected pairs, are shown in the top left.

c. Distribution of the 10-kb LEPAE score. Forty bins ranging from 0 to 1 with increments of 0.025 were used.

A 1-kb version of this figure is in **Figure 3**.

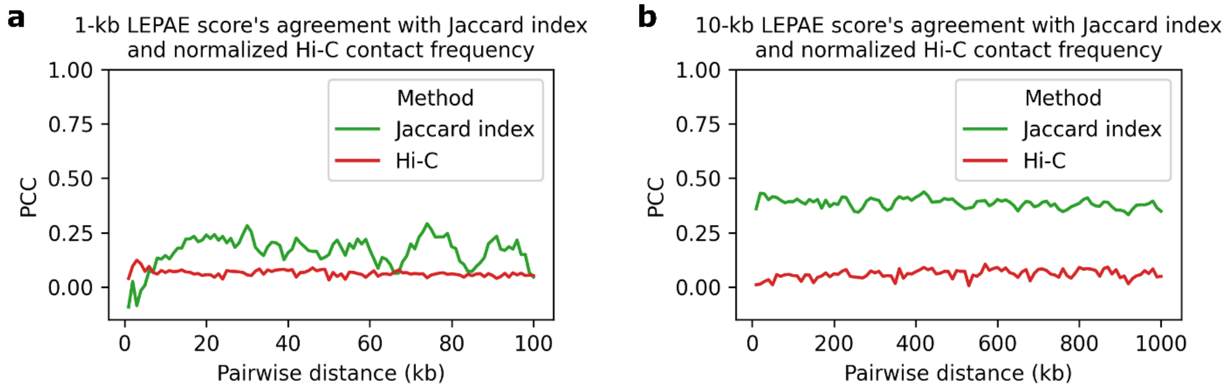

Supplementary Figure 2. LEPAE score's relationship to Hi-C contact frequency and Jaccard index between input features

a. Shown for each pairwise distance (*x*-axis) is PCC of the LEPAE score with either Jaccard index (green) or normalized Hi-C contact frequency (red) for pairs of windows with the specified distance between them. Values belonging to the same method are connected by piecewise linear interpolation.

b. Same as **a** but for 10-kb LEPAE score instead of 1-kb LEPAE score.

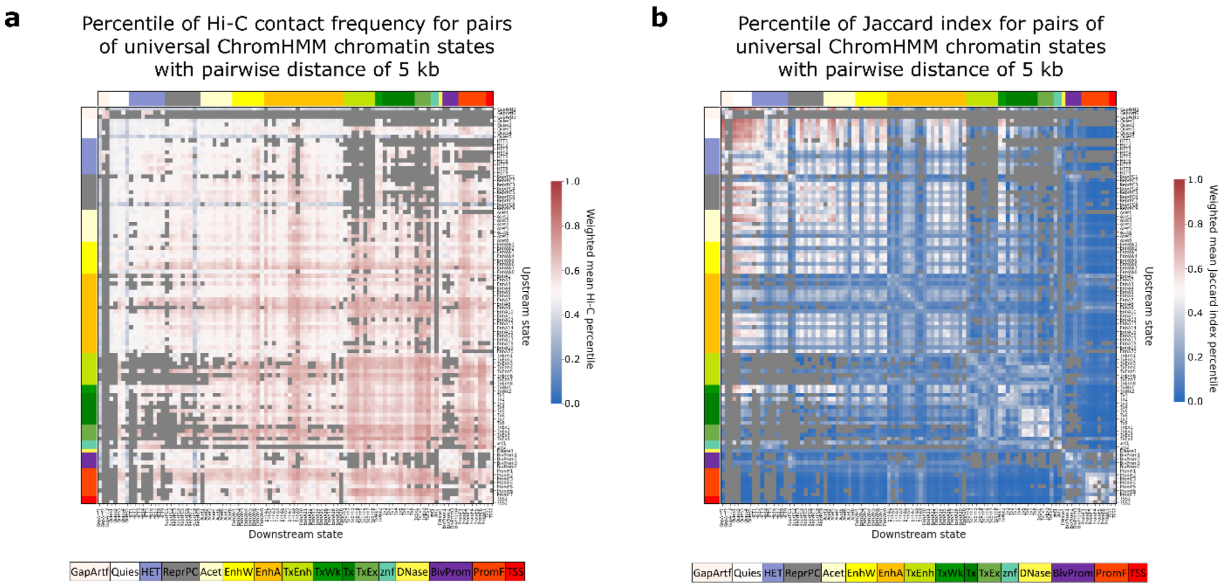

Supplementary Figure 3. Heatmaps of mean percentile of 1-kb Hi-C contact frequency and Jaccard index for pairs of chromatin states with pairwise distance of 5 kb

a. Each cell in the heatmap corresponds to a state pair, one annotating the upstream window of a pair of 1-kb windows (row) and the other annotating the downstream 1-kb window of the same pair (column) with their pairwise distance fixed to 5 kb. The states are from a universal chromatin state annotation based on more than 1000 epigenomic datasets from more than 100 cell or tissue types (Vu and Ernst 2022)(Vu and Ernst 2022). The ordering of states in the rows and columns is the same. Color shown next to the topmost row or leftmost column corresponds to the state group of each state along the column or row, respectively, according to the legend on the bottom left. Colors shown in the cells correspond to a weighted mean Hi-C contact frequency percentile of pairs of windows that are 5 kb apart and are annotated by the states specified in the row and column (**Supplementary Methods**). Color legend for the score is shown on the right. A similar version of this figure but for LEPAE score is in **Figure 5a**.

b. Similar to **a** but with each cell showing percentile based on Jaccard index instead of LEPAE score

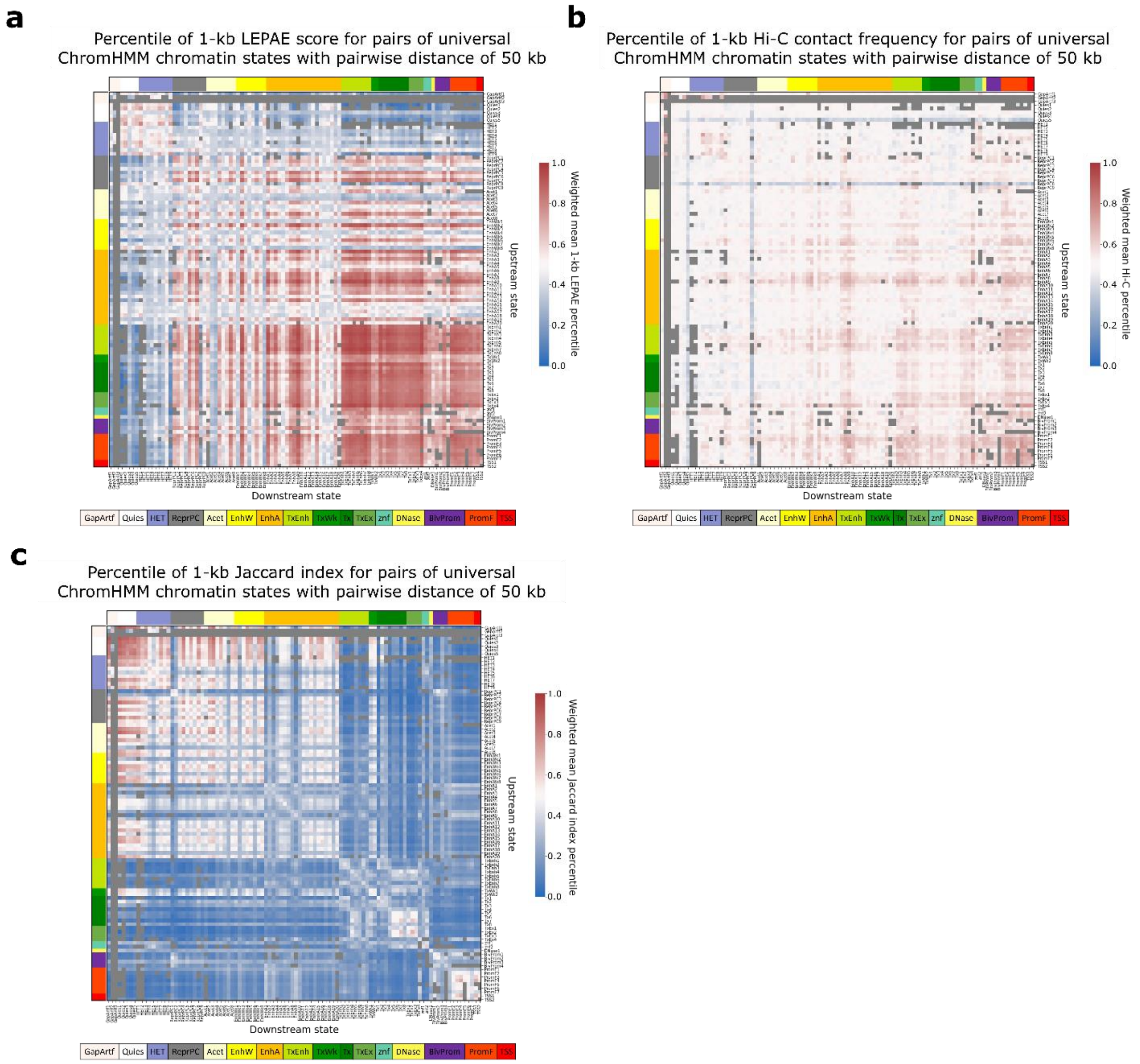

Supplementary Figure 4. Heatmaps of mean percentile of 1-kb LEPAE score, Hi-C contact frequency, and Jaccard index for pairs of chromatin states with pairwise distance of 50 kb

a. Each cell in the heatmap corresponds to a state pair, one annotating the upstream window of a pair of 1-kb windows (row) and the other annotating the downstream 1-kb window of the same pair (column) with their pairwise distance fixed to 50 kb. The states are from a universal chromatin state annotation based on more than 1000 epigenomic datasets from more than 100 cell or tissue types (Vu and Ernst 2022)(Vu and Ernst 2022). The ordering of states in the rows and columns is the same. Color shown next to the topmost row or leftmost column corresponds to the state group of each state along the column or row, respectively, according to the legend on the bottom left. Colors shown in the cells correspond to a weighted mean LEPAE score percentile of pairs of windows that are 50 kb apart and are annotated by the states specified in the row and column (**Supplementary Methods**). Color legend for the score is shown on the right. A similar version of this figure but for pairwise distance of 5 kb is in **Figure 5a**.

b. Similar to **a** but with each cell showing percentile based on Hi-C contact frequency instead of LEPAE score

c. Similar to **a** but with each cell showing percentile based on Jaccard index instead of LEPAE score

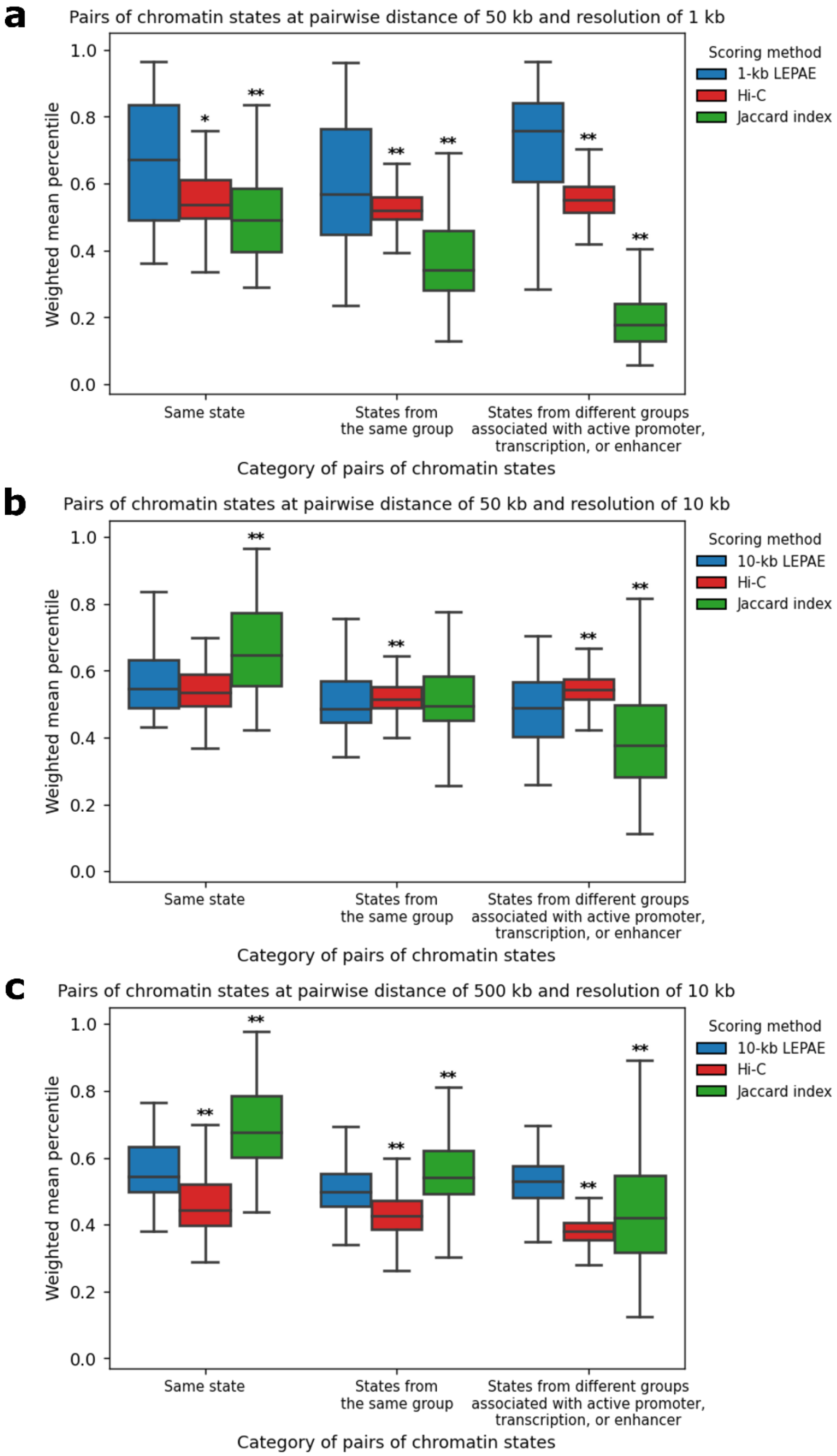

Supplementary Figure 5. Distribution of weighted mean percentile of different scores for pairs of chromatin states.

a. Shown for three different categories of pairs of ChromHMM chromatin states are the distribution of the weighted mean percentile of the 1-kb LEPAE score, Hi-C contact frequency, or Jaccard index, at a fixed pairwise distance of 50 kb as done in **Supplementary Fig. 4**. Among the three categories, the first category shown on the leftmost position on the x-axis corresponds to pairs of the same state. The second, shown in the middle, corresponds to pairs of different states belonging to the same state group. The rightmost category corresponds to pairs of states where both states are associated with active promoters, transcription, or enhancers but are from different state groups. These three categories of pairs of states do not overlap with each other. Within each category, the distributions of the weighted mean percentile of the 1-kb LEPAE score, Hi-C contact frequency, and Jaccard index are shown in blue, red, and green, respectively, according to the score legend on the right. One asterisk and two asterisks above a distribution denotes that there is a significant difference between it and the distribution of weighted 1-kb LEPAE score percentiles within the same category based on a Mann-Whitney U test for p-values less than 0.001 and 0.0001, respectively. Similar versions of this figure but for pairwise distance of 5 kb is shown in **Figure 5b**.

b. Similar to **a** but showing results from 10-kb score resolution at a pairwise distance of 50 kb

c. Similar to **a** but showing results from 10-kb score resolution at a pairwise distance of 500 kb

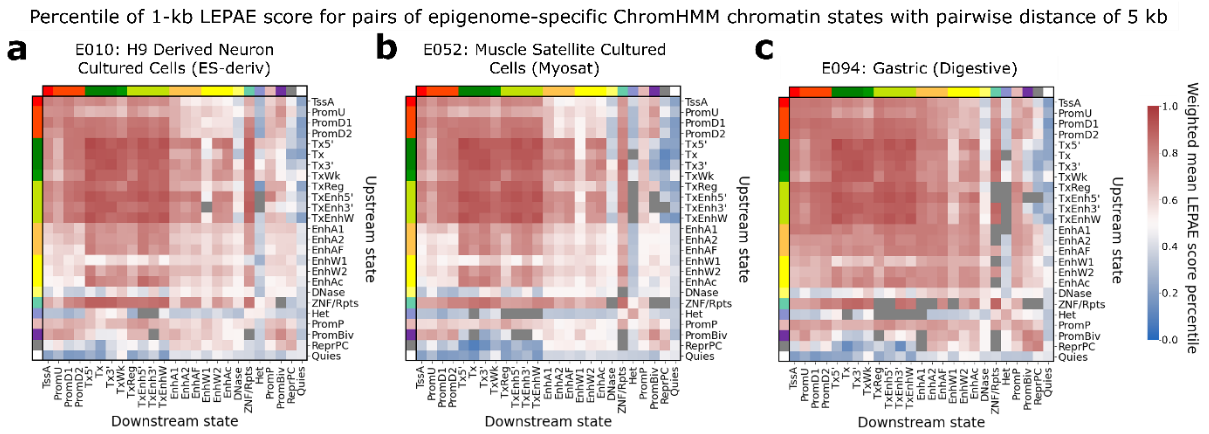

Supplementary Figure 6. 1-kb LEPAE score's relationship to pairs of epigenome-specific chromatin states with pairwise distance of 5 kb.

a. Each cell in the heatmap corresponds to a state pair, one annotating the upstream window of a pair of 1-kb windows (row) and the other annotating the downstream 1-kb window (column) with their pairwise distance fixed to 5 kb. The states are from an epigenome-specific chromatin state annotation from the Roadmap Epigenomics Project (Roadmap Epigenomics Consortium et al. 2015)(Roadmap Epigenomics Consortium et al. 2015), specifically for epigenome with ID E010, Standardized Epigenome Name "H9 Derived Neuron Cultured Cells", and Group "ES-deriv" according to the project's metadata. State ordering in the rows and columns is the same. Color shown next to the topmost row or leftmost column corresponds to the color assigned to each state along the column or row, respectively. Colors shown in the cells correspond to a weighted mean LEPAE score percentile of pairs of windows that are 5 kb apart and are annotated by the states specified in the row and column (**Supplementary Methods**). Color legend for the score is shown on the right end of the figure. Pairs of states that together cover less than 0.0005% of all the bases annotated by pairs of states were excluded from the analysis with grey shown in their corresponding cells.

b. Similar to **a** but for epigenome with ID E052, Standardized Epigenome Name "Muscle Satellite Cultured Cells", and Group "Myosat"

c. Similar to **a** but for epigenome with ID E094, Standardized Epigenome Name "Gastric", and Group "Digestive"

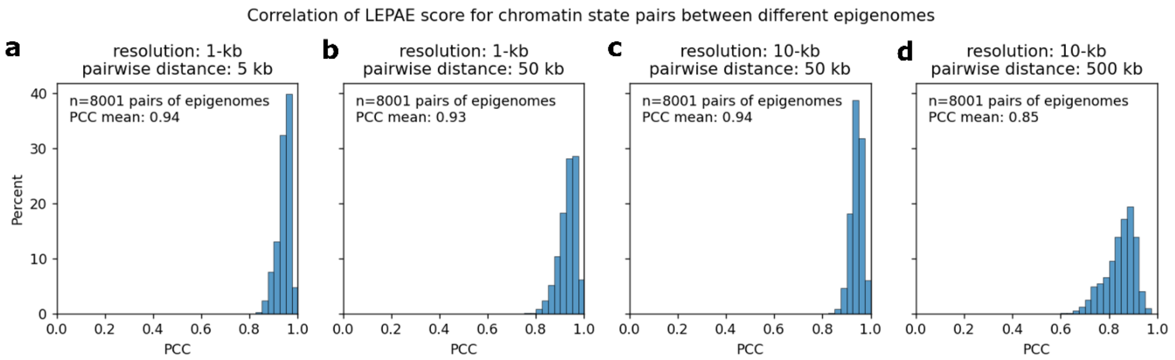

Supplementary Figure 7. Correlation of LEPAE score for chromatin state pairs between different epigenomes.

a. Distribution of PCCs of weighted mean 1-kb LEPAE score percentile of every pair of epigenome-specific chromatin states for genomic windows with pairwise distance of 5 kb computed for every pair of epigenomes. This is based on the 25-state epigenome-specific chromatin state annotation for 127 reference epigenomes from the Roadmap Epigenomics Project. Forty bins from 0 to 1 with increments of 0.025 were used. The number of PCCs in the distribution and their mean are shown in the top left corner.

b. Similar to **a** but for pairwise distance of 50 kb

c. Similar to **a** but for 10-kb LEPAE score and pairwise distance of 50 kb

d. Similar to **a** but for 10-kb LEPAE score and pairwise distance of 500 kb

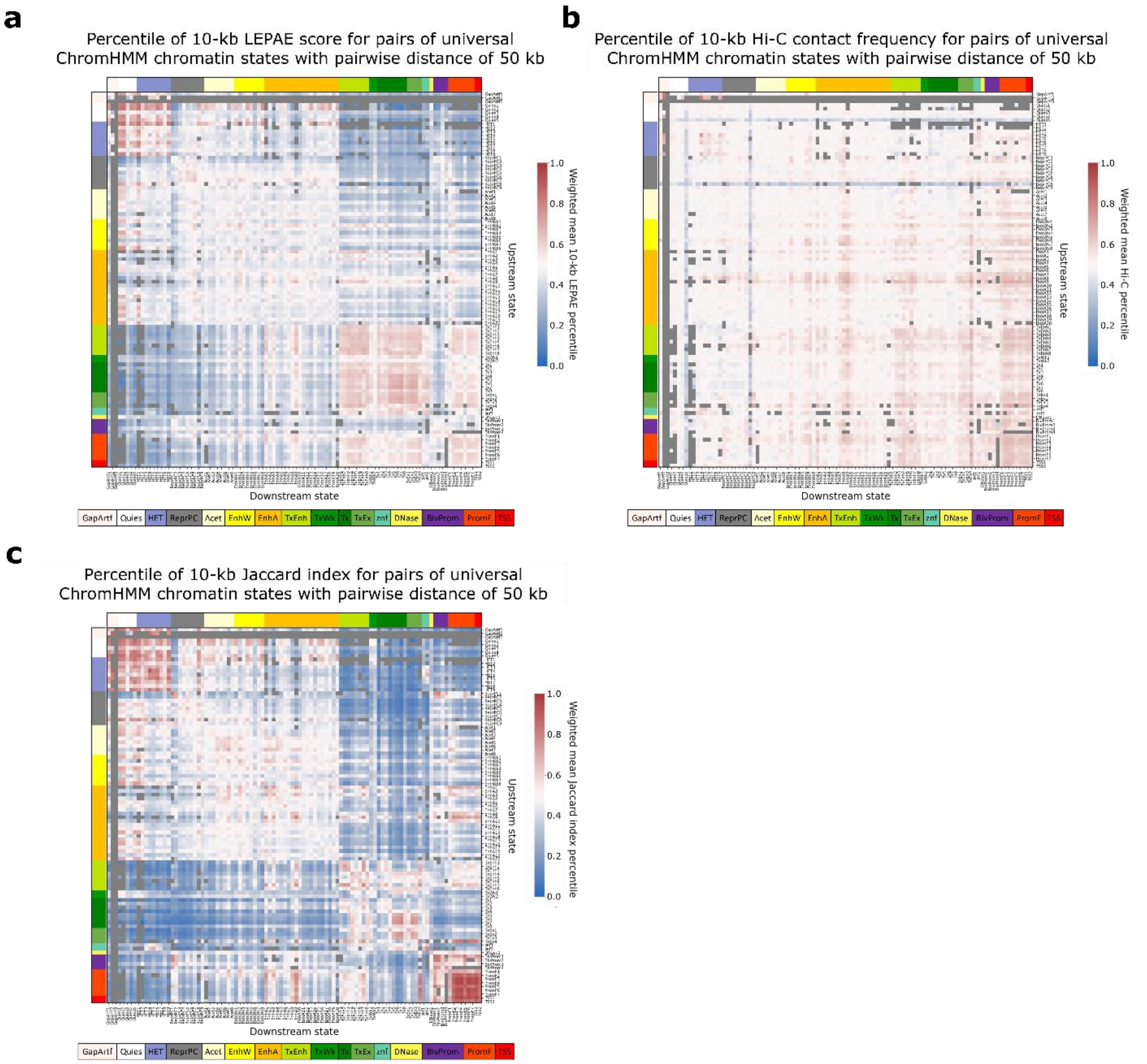

Supplementary Figure 8. Heatmaps of mean percentile of 10-kb LEPAE score, Hi-C contact frequency, and Jaccard index for pairs of chromatin states with pairwise distance of 50 kb

Similar to **Supplementary Fig. 4** but showing results from analyzing at a 10-kb score resolution and with pairwise distance of 50 kb

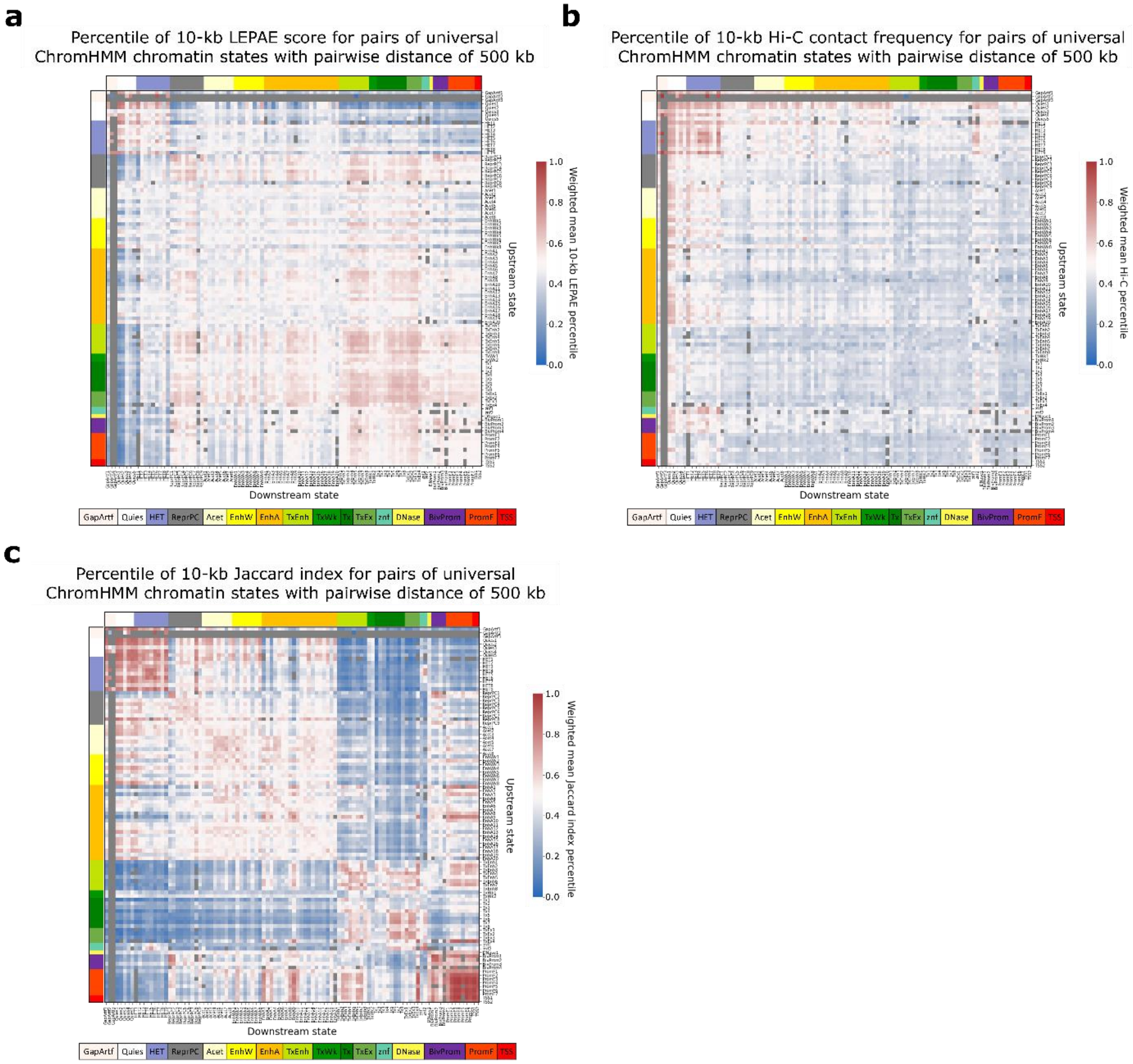

Supplementary Figure 9. Heatmaps of mean percentile of 10-kb LEPAE score, Hi-C contact frequency, and Jaccard index for pairs of chromatin states with pairwise distance of 500 kb

Similar to **Supplementary Fig. 4** but showing results from analyzing at a 10-kb score resolution and with pairwise distance of 500 kb

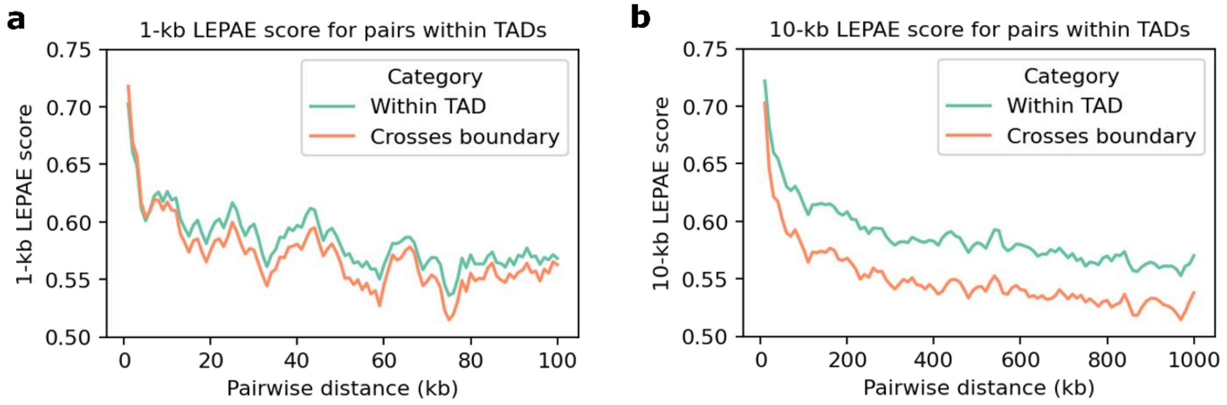

Supplementary Figure 10. LEPAE score's relationship to TAD annotations

a. Shown for each pairwise distance (x-axis) is the mean 1-kb LEPAE score for pairs of windows located within a topologically associating domain (TAD) (turquoise) or the mean score for pairs of windows crossing a TAD boundary (peach). Values belonging to the same category are connected by piecewise linear interpolation.

b. Similar to **a** but for 10-kb LEPAE score

Supplementary Methods

Decision tree evaluation

We trained, applied, and evaluated an ensemble of decision trees using the same procedure described for the ensemble of neural networks (**Methods**), except we used a decision tree in place of a neural network. We also did hyper-parameter selection as done for the ensemble of neural networks, but for the following set of hyper-parameters unique to decision trees:

- Maximum tree depth: 16, 32, 64, 128, 256
- Minimum fraction of samples required to split at an internal node: 0.0005, 0.001, 0.002, 0.005, 0.01
- Minimum fraction of samples required to be at a leaf node: 0.0005, 0.001, 0.002, 0.005, 0.01

The maximum number of features to consider when looking for the best split was set to square root of the total number of features. As done with neural networks, 10 decision trees were ensembled. We used Scikit-learn (version 0.19.1)(Pedregosa et al. 2011)(Pedregosa et al. 2011) for implementation.

Computing Jaccard index

For each pair, given its two binary input feature vectors, we defined *A* as the set of features set to 1 in the first feature vector and *B* as the set of feature set to 1 in the other feature vector. The Jaccard index between the two vectors was defined as:

$$J(A,B) = \frac{|A \cap B|}{|A \cup B|} \qquad (1)$$

If the denominator was zero, the pair was eliminated from our analysis.

Hi-C data

We downloaded in situ Hi-C data for GM12878 (experiment 4DNES3JX38V5) (Rao et al. 2014)(Rao et al. 2014) from the 4DN Nucleome Data Portal (Reiff et al. 2022)(Reiff et al. 2022) from https://4dn-open-data-public.s3.amazonaws.com/fourfront-webprod/wfoutput/a98ca64a-861a-4a8c-92e9-586af457b1fb/4DNFI1UEG1HD.hic. Within the downloaded file, we specifically used values with square root of vanilla coverage (VC_SQRT) normalization applied. We used software straw (Durand et al. 2016)(Durand et al. 2016) to extract the values for the pairs of our interest from the file. If no data was found for a pair in the file, we discarded the pair from our analysis. We downloaded TAD coordinates for GM12878 provided by the 3D Genome Browser (Wang et al. 2018)(Wang et al. 2018) at http://3dgenome.fsm.northwestern.edu/downloads/hg38.TADs.zip.

Computing mean LEPAE score percentile for pairs of chromatin states

For each state pair, s_u and s_d, its mean LEPAE score percentile was computed as follows. First, to make results comparable with the Hi-C contact frequency and the Jaccard index, the percentile of every score value was computed among pairs of windows with all three scores available. Then, for each pair of windows, w_u and w_d, with at least some portion annotated by states s_u and s_d, respectively, the product of the fraction of bases in window w_u annotated by state s_u and the

fraction of bases in window w_d annotated by state s_d was computed. The state pair's LEPAE score percentile for the window was then multiplied by this product. The overall mean of the state pair was the sum of these weighted score percentiles from all applicable pairs of windows divided by the sum of the products of fractions of bases. We excluded from the analysis pairs of states that together cover less than 0.0005% of pairs of bases. For the analysis with universal chromatin states, states associated with active transcription, promoters, or enhancers were defined as states belonging to the following state groups, according to how they are annotated in Vu and Ernst 2022Vu and Ernst 2022: EnhA, PromF, Tx, TxEx, TxEnh, TSS.

Gene analysis

For each protein-coding gene from GENCODE gene annotation (V38) (Frankish *et al.* 2021) with length *l*, we set quartile length *q* to *l* divided by 4. For genes on the positive strand, given the position of the transcription start site (TSS) and transcription end site (TES) of the gene, s_1 and s_2, we defined three sets of bases for the gene as follows:

- Upstream of TSS: s_1 - 4q, s_1 - 3q, s_1 - 2q, s_1 - q
- Within gene: s_1, s_1 + q, s_1 + 2q, s_1 + 3q, s_2
- Downstream of TES: s_2 + q, s_2 + 2q, s_2 + 3q, s_2 + 4q

For genes on the negative strand, the procedure above was reversed such that a distance was subtracted instead of added and vice versa.

We then defined pairs of these bases where at least one base in the pair is within the gene (e.g. s_1 - 3q vs. s_1 + q) and with a maximum pairwise distance of four quartiles. We then compared two pairs that had the same pairwise distance and shared a base within the gene but one pair crossed a gene boundary, TSS or TES, while the other pair did not (e.g. s_1 - q vs. s_1 + q compared to s_1 + q vs. s_1 + 3q). This resulted in 12 pairwise comparisons of pairs of bases for each protein-coding gene. The LEPAE score for a pair of bases was the score of the two 1-kb windows that overlap the bases. This excluded pairs of bases less than 1 kb apart from the analysis.

This procedure was repeated for each protein-coding gene. It allowed us to evaluate whether the LEPAE score, Jaccard index, or Hi-C contact frequency favors pairs of windows within genes over those crossing gene boundaries. Using quartile lengths of each gene rather than a fixed pairwise distance allowed us to control for varying gene lengths.

Fine-mapping analysis

We obtained fine-mapped variants for 94 UK Biobank traits from https://www.finucanelab.org/data(Kanai *et al.* 2021, 2022) and mapped the coordinates to hg38 using liftOver. For a pair of variants in windows *i*, *j* where we assume *i* < *j* and they are *d* bases apart, we added as controls pairs of positions corresponding to (*i - d, i*) and (*j*, *j + d*). We conducted the analysis at 1-kb resolution considering variants within 100 kb and also at 10-kb resolution considering pairs of variants within 1 Mb. For each resolution, we separately analyzed the fine-mappings based on FINEMAP (Benner *et al.* 2016) and SuSiE (Wang *et al.* 2020). For each resolution and fine-mapping method, we computed the average LEPAE score for the original pairs of variants and control pairs for each of the 94 traits and determined which average was greater. We repeated the analysis for the GM12878 Hi-C contact frequency and Jaccard Index.

References

Benner C, Spencer CCA, Havulinna AS *et al.* FINEMAP: Efficient variable selection using summary data from genome-wide association studies. *Bioinformatics* 2016;32, DOI: 10.1093/bioinformatics/btw018.

Durand NC, Robinson JT, Shamim MS *et al.* Juicebox Provides a Visualization System for Hi-C Contact Maps with Unlimited Zoom. *Cell Syst* 2016;3:99–101, DOI: 10.1016/j.cels.2015.07.012.

Frankish A, Diekhans M, Jungreis I *et al.* GENCODE 2021. *Nucleic Acids Res* 2021;49:D916–23, DOI: 10.1093/nar/gkaa1087.

Kanai M, Elzur R, Zhou W *et al.* Meta-analysis fine-mapping is often miscalibrated at single-variant resolution. *Cell Genomics* 2022;2, DOI: 10.1016/j.xgen.2022.100210.

Kanai M, Ulirsch JC, Karjalainen J *et al.* Insights from complex trait fine-mapping across diverse populations. *medRxiv* 2021.

Pedregosa F, Gramfort A, Michel V *et al.* Scikit-learn : Machine Learning in Python. *Journal of Machine Learning Research* 2011.

Rao SSP, Huntley MH, Durand NC *et al.* A 3D Map of the Human Genome at Kilobase Resolution Reveals Principles of Chromatin Looping. *Cell* 2014;159:1665–80, DOI: 10.1016/j.cell.2014.11.021.

Reiff SB, Schroeder AJ, Kırlı K *et al.* The 4D Nucleome Data Portal as a resource for searching and visualizing curated nucleomics data. *Nat Commun* 2022;13:2365, DOI: 10.1038/s41467-022-29697-4.

Roadmap Epigenomics Consortium, Kundaje A, Meuleman W *et al.* Integrative analysis of 111 reference human epigenomes. *Nature* 2015;518:317, DOI: 10.1038/nature14248.

Vu H, Ernst J. Universal annotation of the human genome through integration of over a thousand epigenomic datasets. *Genome Biol* 2022;23:9, 10.1186/s13059-021-02572-z.

Wang G, Sarkar A, Carbonetto P *et al.* A simple new approach to variable selection in regression, with application to genetic fine mapping. *J R Stat Soc Series B Stat Methodol* 2020;82, DOI: 10.1111/rssb.12388.

Wang Y, Song F, Zhang B *et al.* The 3D Genome Browser: A web-based browser for visualizing 3D genome organization and long-range chromatin interactions. *Genome Biol* 2018;19, DOI: 10.1186/s13059-018-1519-9.
